# Direct-write orientation of charge-transfer liquid crystals enables polarization-based coding and encryption

**DOI:** 10.1038/s41598-020-72037-z

**Published:** 2020-09-18

**Authors:** Madeline Van Winkle, Harper O. W. Wallace, Niquana Smith, Andrew T. Pomerene, Michael G. Wood, Bryan Kaehr, Joseph J. Reczek

**Affiliations:** 1 Department of Chemistry, Denison University, Granville, OH 43023 USA; 2 Sandia National Laboratories, Albuquerque, NM 87185 USA; 3 Center for Integrated Nanotechnologies, Sandia National Laboratories, Albuquerque, NM 87185 USA

**Keywords:** Self-assembly, Information storage, Liquid crystals

## Abstract

Optical polarizers encompass a class of anisotropic materials that pass-through discrete orientations of light and are found in wide-ranging technologies, from windows and glasses to cameras, digital displays and photonic devices. The wire-grids, ordered surfaces, and aligned nanomaterials used to make polarized films cannot be easily reconfigured once aligned, limiting their use to stationary cross-polarizers in, for example, liquid crystal displays. Here we describe a supramolecular material set and patterning approach where the polarization angle in stand-alone films can be precisely defined at the single pixel level and reconfigured following initial alignment. This capability enables new routes for non-binary information storage, retrieval, and intrinsic encryption, and it suggests future technologies such as photonic chips that can be reconfigured using non-contact patterning.

## Introduction

The ability to generate and distinguish polarized light has widespread utility—from animals that have evolved dichroic polarizers to orient to their surroundings using optical compasses^1^, to various modern technologies including forensic instruments, photonic integrated circuits, and ubiquitous digital displays. In its simplest incarnation, a polarizer is an anisotropic arrangement of material, such as a grid of parallel wires^2^. For polarizers operating at visible wavelengths, atoms, molecules or nanoparticles are aligned on a surface or in a film, selectively absorbing or reflecting orientations of light that are parallel to their alignment. This common configuration is used for the majority of polarizing films found in sunglasses and window coatings and, in general, is used to produce relatively large area static films. For example, in typical liquid crystal displays (LCDs), static cross-polarizers sandwich an electronically addressable liquid crystal layer^2^. In this case, the liquid crystals (LCs) become polarized in the presence of an electric field, leading to LC alignment and pixel interpretation (i.e., on/off, grayscale) that is contingent on the properties (size, shape, switching speed) of the underlying patterned electrodes. However, if polarization and LC alignment could be continuously varied in the absence of these constraints, a single pixel could provide a grayscale transmission response under a set illumination, dramatically increasing the information storage capacity from binary (1 bit) to continuously variable (analog).

Changing the orientation of a polarizer at the scale of a ‘pixel’ (~ tens of microns) is not generally considered given the fabrication approaches for polarized films and optics and the geometric constraints of planar electrodes used to control LC orientation in devices^3^. Thus grayscale transmission (e.g., for optical encryption) requires multiple LC layers, optical elements and polarizers to interact along the optical path^4–11^. Advances in nano-scale periodic materials for precise control of electromagnetic propagation (i.e., metasurfaces^12^) can enable on-chip polarization control, though typically with narrow bandwidth and limited ability for reconfiguration or control of angle, with few exceptions^13–15^. Wire-grid type polarizers built from polymers^16,17^ nanotubes^18^, nanowires^19^, and etched metal gratings^20^ improve performance beyond traditional polarized films (derived from H-sheet polarizers^21^) but use bulk/large area approaches for alignment. Local alignment of LCs can be achieved with optical resolution by photo-aligning substrates that dictate the pre-tilt angle^22–24^ and with site-specific accuracy using direct laser writing^25,26^, but these approaches do not afford reconfigurability.

**Figure 1:**
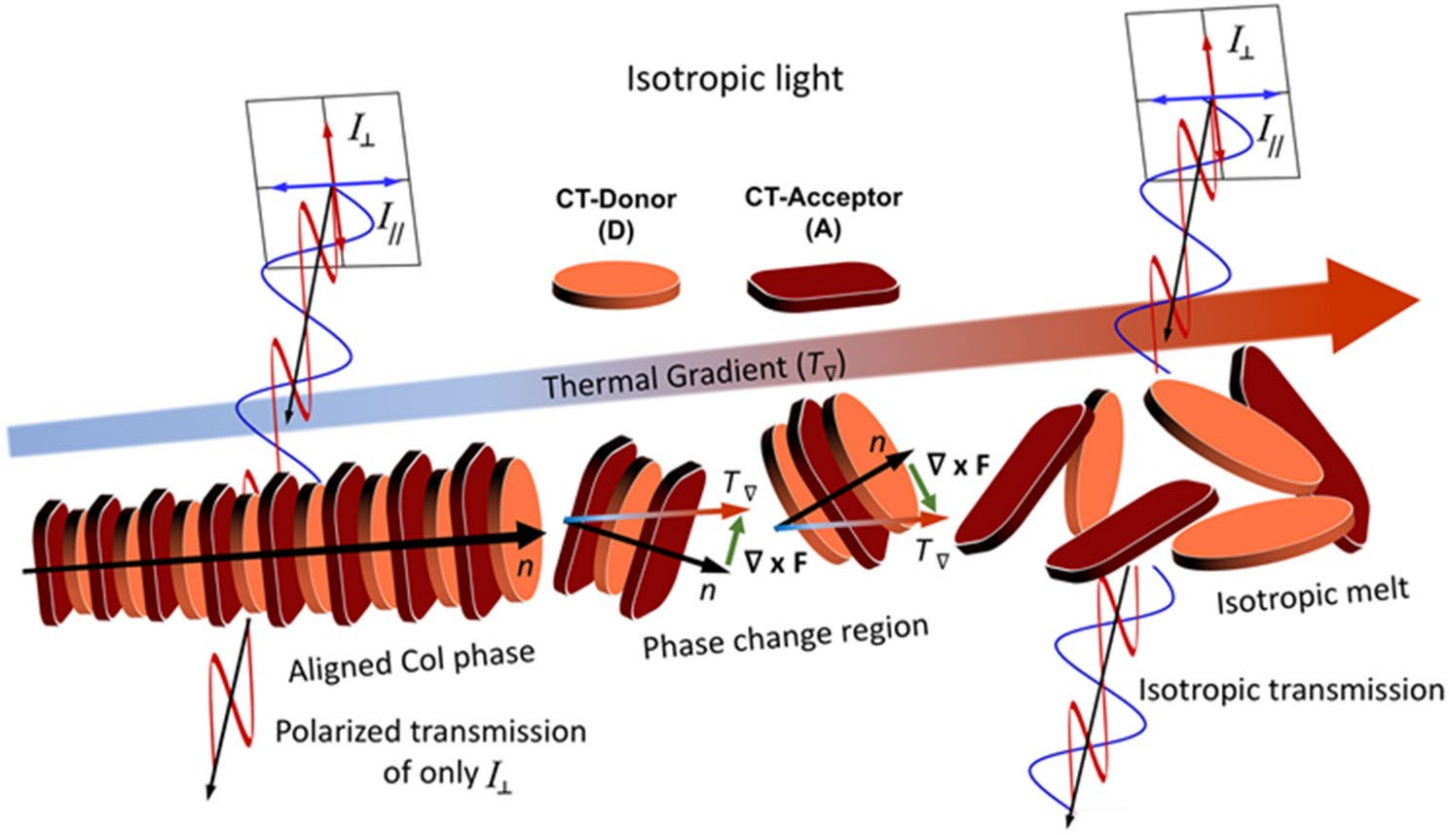
Light polarization by aligned DACLC regions. Fine control of columnar alignment via laser-induced thermal gradient (left) from isotropic light transmission through unaligned (melted) DACLC regions.

Recently we demonstrated the ability to locally orient the alignment of a class of charge-transfer liquid crystals referred to as donor acceptor columnar liquid crystals (DACLCs) using a laser direct-write technique^27^. When cooled slowly from a melt, these donor/acceptor constituents self-assemble to form strongly dichroic, rod-like stacks (Fig. 1). By directionally controlling this melt/cool response using a scanning laser passing over a DACLC film, the irradiated region can be rendered isotropic (with rapid cooling) or strongly dichroic (with slower cooling). The latter induces columnar order by generating a thermal gradient (*T*_*∇*_) which acts on the columnar director (n) of a cooling DACLC region to align the material in the plane of *T*_*∇*_ (i.e., the direction of laser writing; Fig. 1). The characteristic charge-transfer (CT) absorption seen in bi-component donor–acceptor materials is observed only in the direction of π–π stacking; thus aligned DACLCs can act as highly anisotropic absorbers of light oriented parallel to columnar alignment, often achieving dichroic ratios (α_⫽_/α_⊥_) > 30 in the CT region^28^. In principle, the relative angle of aligned columns can be arbitrarily dictated using this approach simply by adjusting the direction of the thermal gradient.

Here we consider this possibility to achieve precise control over the angle of columnar alignment in DACLC films and the corresponding polarization of transmitted light. This optically written molecular assembly allows for regions with discrete polarization that can be distinguished on a micron-scale. The practical relevance of this molecular system is illustrated through the optical writing and reading of images and data in DACLC films. Importantly, the described alignment technique results in patterned areas that are easily re-written using a non-contact (e.g., electric field independent) approach, retain optical functionality as standalone films, and have synthetically tunable rates of degradation, providing independent control over the retention time of stored information. Further, the analog response of the system increases the density of information retrieved from a coded pixel compared to traditional digital methods, while also illustrating new modes of passive data encryption.

## Results and discussion

### Patterned control of light polarization

**Figure 2:**
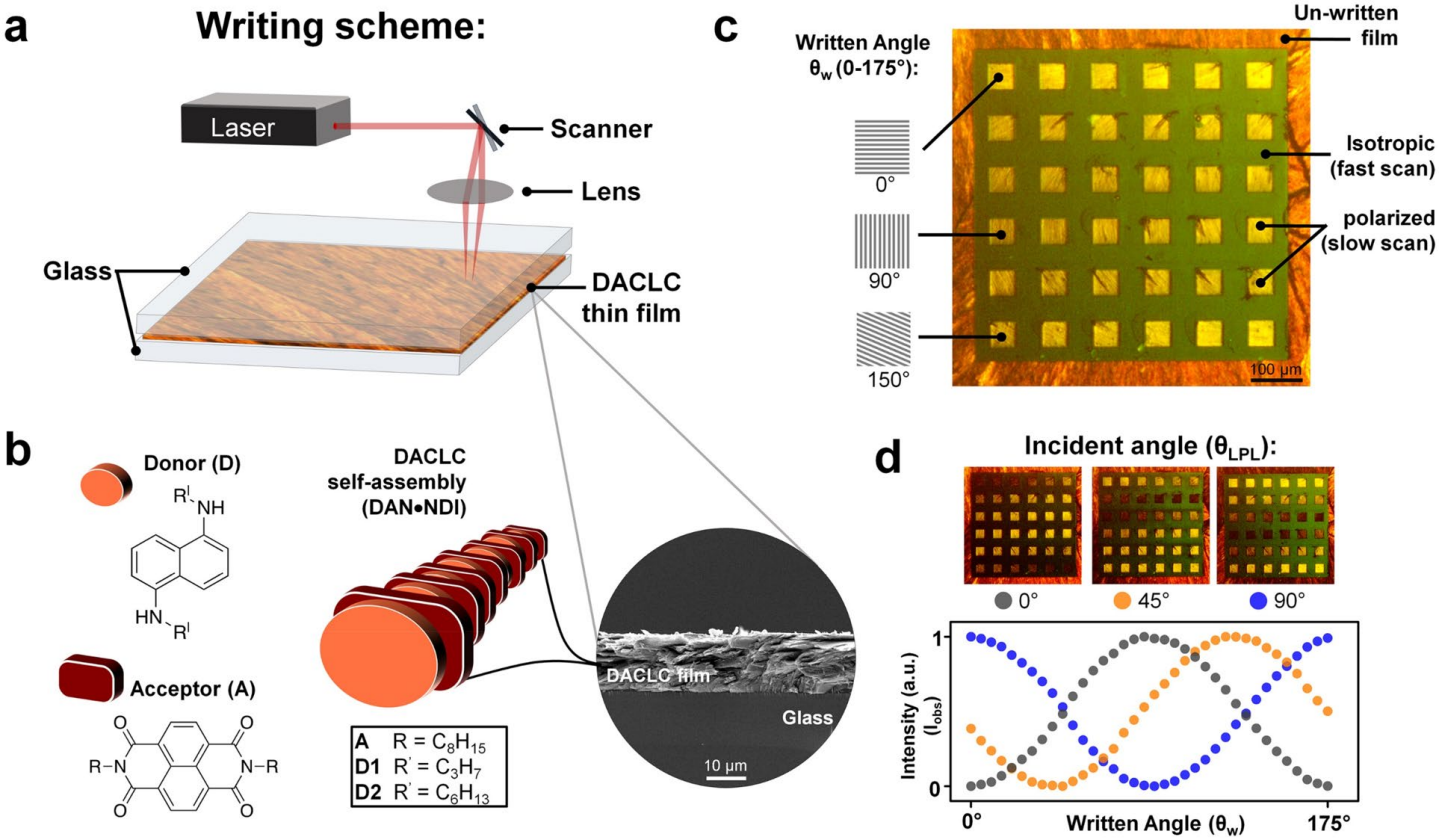
Laser-direct-write of DACLC films. (**a**) Schematic of laser writing setup. A 780 nm beam is focused into a thin (12–20 µm) DACLC film to induce melting at the point of focus. (**b**) Diagram of columnar phase stacking of alternating DAN·NDI molecules, and SEM cross-section of a film. (**c**) DACLC film patterned with a 6 × 6 grid of 50 × 50 µm regions aligned at 5° increments from 0° to 175° over an isotropic background (imaged using unpolarized light). (**d**) M-LPI images of patterned film from (**c**) taken using θ_LPL_ = 0°, 45°, and 90°. Intensity of LPL transmitted (I_obs_) through each aligned region is plotted as a function of the region’s angle of columnar alignment (θ_w_) in accordance to Eq. 1 (R^2^ > 0.97).

DACLC films were sandwiched between glass substrates and mounted into a scanning laser setup (Fig. 2a). Two different materials were characterized, each consisting of the *N,N′*-octyl naphthalenediimide acceptor with either 1,5-di-propyl-aminonapthalene (A·D1) or 1,5-di-hexyl-aminonapthalene (A·D2) (Fig. 2b). To examine the correlation between desired alignment of patterned DACLC films and the real polarization of transmitted light, an isotropic square was laser-patterned followed by re-writing areas within the square resulting in a 6 × 6 grid of anisotropic regions aligned in intended increments of 5° (Fig. 2c). Importantly, any region in the film can be (re)patterned using laser direct write^27^ as well as “reset” by heating the film above the DACLC isotropic transition temperature (~ 160 °C). The resulting grids were imaged using optical microscopy with linearly polarized illumination (M-LPI) with light oriented at 0°, 45°, and 90° (θ_LPL_). Images were analyzed to quantify the intensity of linear polarized light (LPL) transmitted (I_obs_) through each aligned region, plotted as a function of the written angle (θ_w_) (Fig. 2d). A sinusoidal relationship between θ_w_ and I_obs_ is apparent in each plot, exhibiting the same behavior as light passed between two in-line polarizers (θ_p1_ and θ_p2_) according to Eq. 1 (Malus’ law). Substituting θ_LPL_ and θ_w_ for θ_p1_ and θ_p2_ respectively, Malus’ law directly applies to laser-aligned DACLCs where *k* is related to the dichroic ratio of the film and b is a baseline correction due to “dark” transmission (Eq. 2) and illustrates that the laser-aligned DACLC regions effectively act as independently written polarizers.

1 $$ {\text{I}}_{{{\text{obs}}}} = {\text{ I}}_{{\text{I}}} {\cos}^{{2}} (\theta_{{{\text{p1}}}} {-}\theta_{{{\text{p2}}}} ) $$

2 $$ {\text{I}}_{{{\text{obs}}}} = k{\text{I}}_{{\text{I}}} {\cos}^{{2}} (\theta_{{{\text{LPL}}}} {-}\theta_{{\text{w}}} ) \, + {\text{ b}} $$

**Figure 3:**
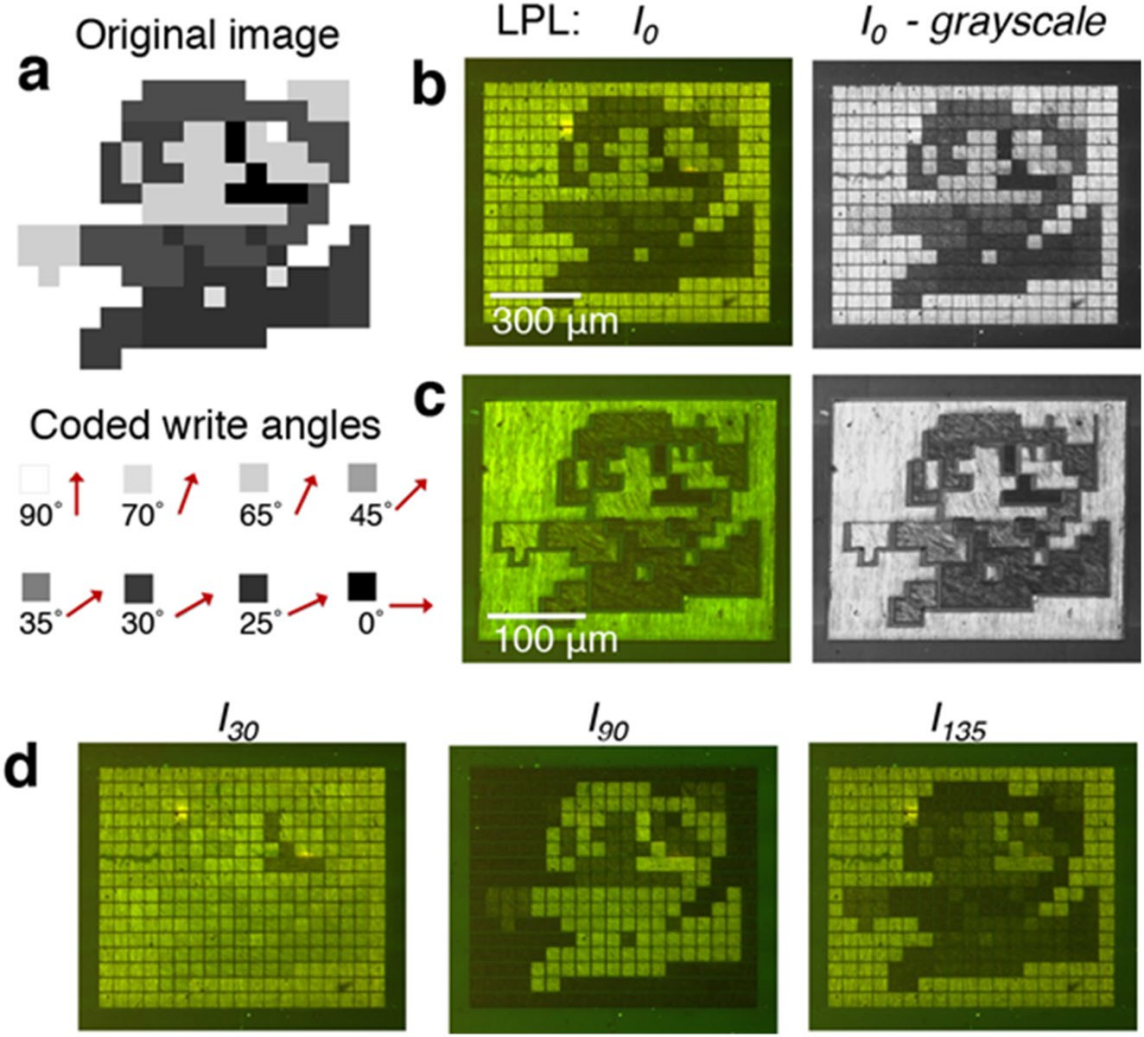
Grayscale image encoded into a DACLC film. (**a**) Target image (top) and write angles corresponding to distinct grayscale values of the image pixels (bottom). M-LPI images of films encoded with (**b**) uniform pixels and (**c**) regional pixels at θ_LPL_ = 0° (shown also in grayscale to demonstrate close similarity to the original image). (**d**) M-LPI images of the pixel-wise encoded film at θ_LPL_ = 30°, 90°, and 135°.

The correlation between written DACLC alignment and resultant LPL transmission can be used to control the relative intensity of DACLC regions with respect to incident LPL angle. This ability to precisely pattern LPL transmission affords a mechanism for inscribing information into a film, correctly interpreted only at an intended θ_LPL_. To explore this concept, we encoded an 8-bit image in a DACLC film, designed to be viewed with a specific θ_LPL_. Each distinct grayscale level of the original image was associated with a unique write angle (θ_w_) based on the predicted LPL transmission through a DACLC region for the chosen θ_LPL_ (Fig. 3a).

Two approaches for optically encoding a grayscale image into a DACLC film are illustrated. The first directly transposes each pixel of the original image to a specific region in the DACLC film, in this case a 50 × 50 µm square (Fig. 3b). Each square is independently written with one of the eight coded alignments. The image is viewed as the compilation of discretely aligned DACLC regions. Alternatively, original image pixels of the same shade can be grouped and patterned as a continuous region on the DACLC film (Fig. 3c). The latter can allow for more complex patterning and smaller feature resolution (< 10 µm) as aligned regions are no longer pixelated and can take any size or form. However, we note that an isotropic border remains visible between neighboring regions, which is attributed to overlap of the laser-scanned regions. As noted above, the encoding of θ_w_ (in this case to represent relative shading) is specific to an intended viewing polarization, θ_LPL_. When either of the two written films are viewed at the intended LPL angle (in this case, θ_LPL_ = 0°), the original image is clearly represented. However, viewing the film at different LPL orientations yields “washed-out” images of poorer contrast (θ_LPL_ = 30°, 135°) or an image with inverted grayscale levels (θ_LPL_ = 90°) (Fig. 3d). In other words, accurate interpretation of the information encoded into the DACLC film is contingent upon viewing with an intended input polarization angle.

Unlike traditional polarizing films, discrete regions of DACLC films can be independently oriented, and re-oriented, down to the micron scale. Considering a case of two overlapping DACLC films, θ_LPL_ and θ_w_ (Eq. 2) is replaced by θ_w1_ and θ_w2_ (Eq. 3), resulting in a unique intercorrelated value of I_obs_ for each pixel.

3 $$ {\text{I}}_{{{\text{obs}}}} = k\prime {\text{I}}_{{\text{I}}} {\cos}^{{2}} (\theta_{{{\text{w1}}}} {-}\theta_{{{\text{w2}}}} ) \, + {\text{ b}}\prime $$

**Figure 4:**
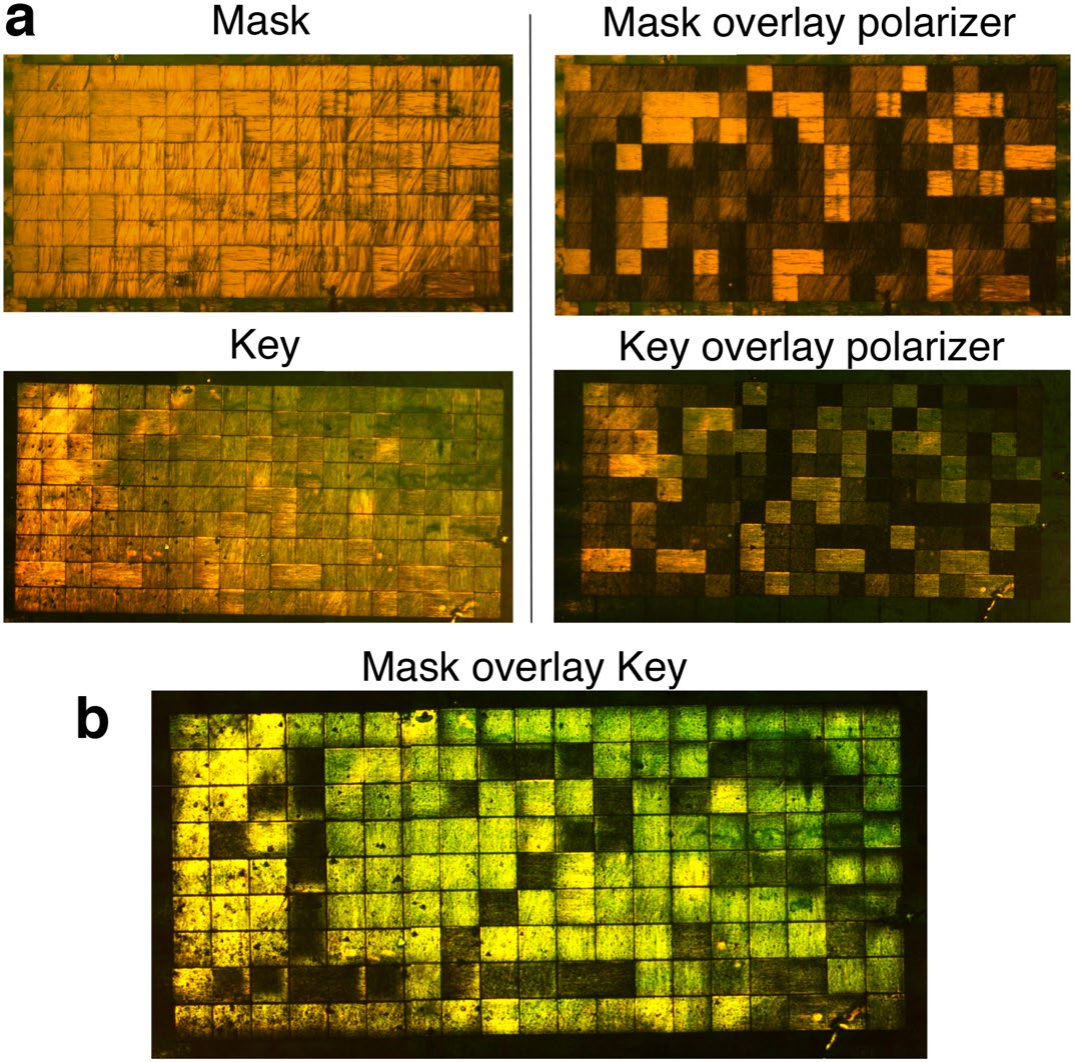
Defining the input polarization angle via a patterned DACLC Mask. (**a**) Microscope images of a DACLC “mask” with 50 × 50 µm pixels written at random values of θ_w_ and a corresponding “key” viewed with unpolarized light (left) and LPL (right). (**b**) Microscope image of overlaid mask and key films.

Together these features allow for encryption schemes that take advantage of overlaid polarizers^4^. As a demonstration, arbitrarily aligned regions are written in a film serving as a “mask”, with a second film, the “key”, written relative to the mask so that information is revealed only upon correct overlay of the two films. On overlay, bright transmission of light corresponds to stacked DACLC regions with similar orientation (θ_w1_ ≈ θ_w2_), while orthogonal regions (θ_w1_ ≈ θ_w2_ + 90°) appear dark. Importantly, no intelligible information can be obtained from either the mask or key films independently, using unpolarized or polarized light (Fig. 4a); the encrypted information is only revealed on mask-key overlay (Fig. 4b). In addition, more than one key can be designed to work with a given mask, meaning that one mask can be used to view different messages (Supplementary Fig. 1). This scheme enables fast and low-cost authentication, useful for documents, currency, supply chains, etc.

### Information resolution, encoding and retrieval in a single DACLC film

The ability to achieve microscopic patterning of polarization provides opportunities for more complex methods of information encoding and retrieval. In place of a key overlay, the relative θ_w_ of written regions can be determined in a single film using M-LPI by fitting I_obs_ at known values of θ_LPL_ using the phase determinant of Eq. 2 (θ_w_ = θ_LPL_ – π/2). Given the periodic relationship between DACLC alignment direction and LPL transmittance, the full span of θ_w_ (0°–180°) can be differentiated by “splining” the measured transmittance intensities from a minimum of only three different LPL images^29^ (Supplementary Fig. 2). While any values of θ_LPL_ can be used, maximum resolution over all possible θ_w_ is achieved comparing I_obs_ at 45° increments (e.g., θ_LPL_ = 0°, 45°, and 90°). Of note, although three images are required to distinguish the maximum range of relative angles 0°–180° (e.g., 45° and 135° would be indistinguishable with only two LPL images), it is possible to distinguish relative θ_w_ between 0°–90° fitting only two images.

**Figure 5:**
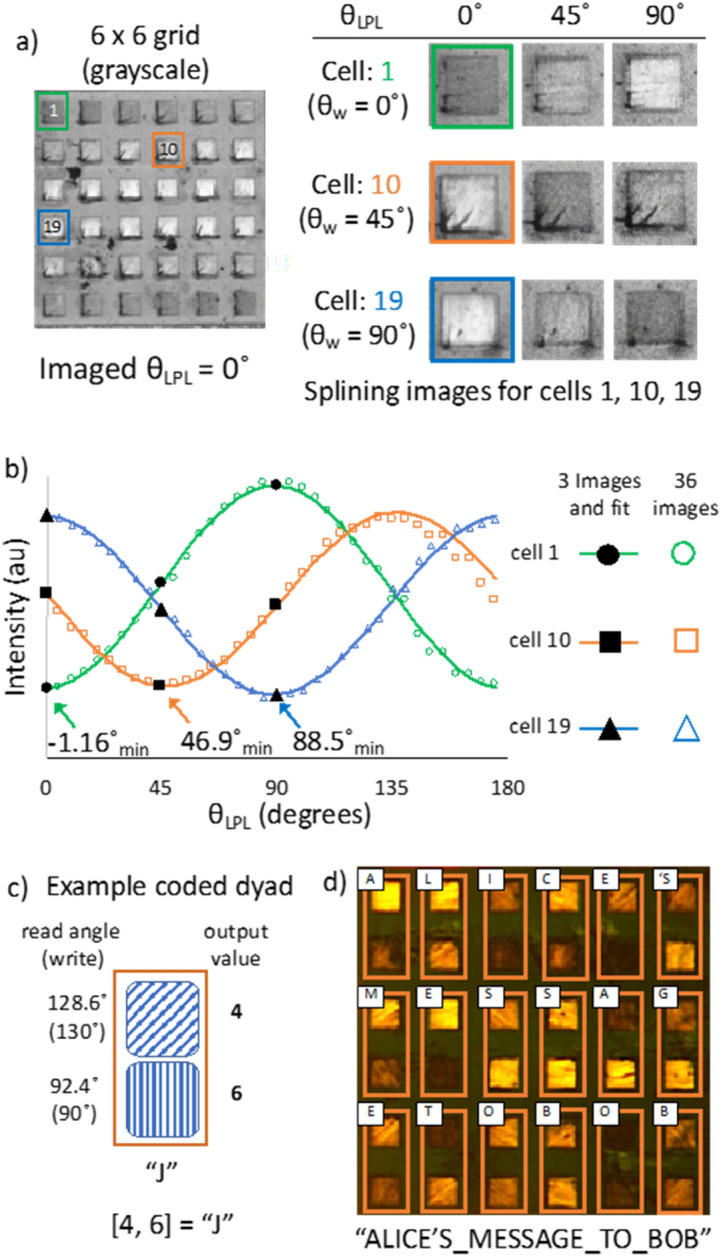
Information encoding and retrieval using DACLC polarizers. (**a**) 36-cell grid imaged in black and white (θ_LPL_ = 0°) and representative LPL images (θ_LPL_ = 0°, 45°, and 90°) used for splining of three individual cells; (**b**) Graph of intensity (I_obs_) vs θ_LPL_ for grid cell 1, 10, and 19. Shows the measured intensity of the splining images in (**a**) (black markers), their three-image fit (solid lines), and an overlay of the measured intensity at 5° intervals (open markers); (**c**) Illustration of dyad encoding scheme; (**d**) Base-10 encoded DACLC film and data retrieval of “ALICE’S_MESSAGE_TO_BOB”.

With this consideration, we interrogated the minimum difference in alignment direction that could be reliably distinguished in patterned DACLC polarizers using the splining technique. Two 6 × 6 grids were prepared with 50 × 50 µm cells aligned in 5° increments from 0° to 175° (Fig. 5a). Considering only three M-LPI images of each 36-cell grid (θ_LPL_ = 0°, 45°, and 90°; Fig. 5b, black markers), the relative direction of DACLC alignment in each of the 72 cells was calculated by the splining technique (Fig. 5b, solid lines). These values were compared to the actual write angles for accuracy (θ_w-calc_ − θ_w_), showing an average difference of − 0.47° ± 2.61° (n = 78). This can be related to an accuracy range with a t-distribution, and yields a predicted accuracy of 93.78%, 99.44%, and 99.97% for θ_w-calc_ within a 10°, 15°, and 20° resolution window respectively. Importantly, the calculated θ_w_ values from splining only three M-LPI images were of negligible difference compared to θ_w_ values calculated with a sine-fit of 36 M-LPI images taken in 5° increments (Fig. 5b, open markers). Thus, retrieval of written alignment angles can be practically achieved with minimal loss in resolution using only three M-LPI images.

To illustrate write-read capability, a data encoding and retrieval process using DACLC grids was designed. With a θ_w_ resolution of 10°, there are 19 possible identities for each cell (i.e., base-19): each of eighteen 10° increments from 0° to 170°, or isotropic. These base-19 “bits” were paired as dyads, each able to store 19^2^ = 361 possible data states. Each dyad was then coded to a text character (Fig. 5c, Supplementary Table 1). The text-based code was used to transcribe a message as a 6 × 6 DACLC grid, which was then independently imaged at θ_LPL_ = 0°, 45°, and 90° to retrieve and decode the message “ALICE’S_MESSAGE_TO_BOB” (Supplementary Table 2). A single error in decoding (C → B) was observed, consistent with the 93.8% accuracy described above for 10° resolution and easily addressed in practice using redundant encoding. To illustrate the available trade-off between storage density and readout accuracy per bit, the same DACLC film was re-interpreted without error using an effective θ_w_ resolution of 20° (i.e., base-10) (Fig. 5d, Supplementary Table 2). Thus, information encoding with this system can use any angle-resolution scheme while considering that increasing retrieval accuracy (using larger angle increments) decreases data capacity per bit.

### Modular molecular composition: “degradation” of CT absorption (and dichroism)

**Figure 6:**
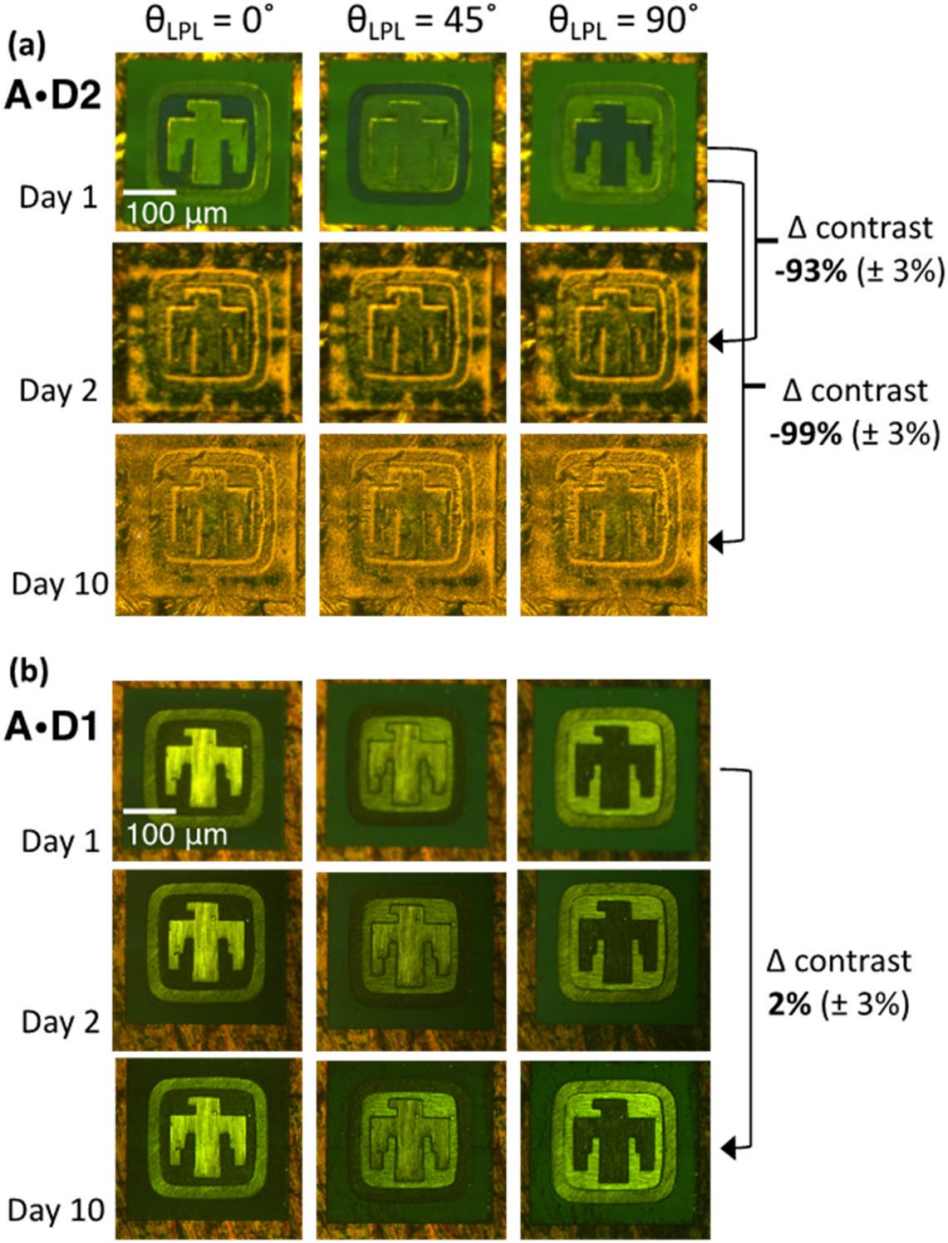
Molecular component-dependent lifetime of encoded information shown in **t**ime-lapse images taken 2 and 10 days after laser-writing of an image on two different DACLC films. (**a**) Film A:D2 showing major loss of optical contrast between written regions (data integrity) in just 2 days, and significant loss of contrast after 10 days under ambient conditions (see Supplementary Fig. 2 for contrast calculation) in comparison to A:D1 (**b**) where no statistical loss of contrast is observed at 10 days.

While most DACLC materials exhibit strong CT absorption in at least one Col phase that is largely independent of the side-chains, the persistence of CT absorption in these materials at room temperature can vary through alteration of side-chain structure^28,30,31^. For example, the dichroic properties of laser-written samples of A:D2 (hexyl chains on the donor) were observed to fade in a matter of days, while samples of A:D1 (propyl chains of donor) maintain dichroic properties indefinitely—shown here using time-lapse imaging of written DACLC films (Fig. 6). Films of A:D2 display high contrast on initial writing, but the ability to distinguish between differently aligned regions by optical transmission diminishes over the course of several days and is lost by day 10 (Fig. 6a). However, the contrast between differently aligned regions in films comprised of A:D1 shows no loss of contrast at 10 days (Fig. 6b) and appears to persist indefinitely when kept under ambient conditions (at the time of this submission, we have observed no alteration of contrast in the A:D1 film for over 18 months). This tunable persistence achieved via molecular design can provide a predictable life-span for information encoded in DACLCs, offering an additional level of security via autonomous self-destruction.

We have described a material set and patterning approach to fabricate complex rewritable optical polarizers using precision laser-scanning. This technique enables adaptable micron-scale control over optical polarization, which can be interrogated using application-specific resolution parameters. This variable functionality lends rewritable DACLC materials to multiple applications including data storage, retrieval and encryption as demonstrated herein. As an additional consideration, fabricating polarization optics in a write/rewrite fashion suggests the possibility to develop a photonic analogue to the field programmable gate array via rewritable waveguiding. Indeed, the measured index contrast between isotropic and patterned regions (∆n = 0.2; Supplementary Fig. 3) is suitable for waveguiding and a feasible approach if patterned on an appropriate substrate (Supplementary Fig. 4). Finally, the ability to chemically build in the lifespan of the patterned material provides additional protection for encoded information and optical functions.

## Methods

### General considerations

All commercial reagents and solvents were obtained from Sigma-Aldrich or Fischer Scientific and were used as purchased without additional purification. Compounds **1** and **2** were synthesized following published procedures.^S1^ ^1^H and ^13^C NMR spectra were collected using a Bruker Avance 400 MHz spectrometer at 25 °C. LPL microscopy was performed using an Olympus BX51TRF microscope and accessories from McCrone Microscopes in transmission mode on a Linkam large area thermal stage. Images were captured with a PAXCAM 3 camera. Thermal analysis data by DSC was collected on a Q20 instrument with an RSC cooling system from TA instruments. UV/Vis spectroscopy was performed on a JAZ-PX spectrophotometer from Ocean Optics. LPL UV/Vis spectroscopy was performed by fitting the JAZ-PX spectrophotometer directly to the Olympus BX51TRF microscope ocular. Variable temperature powder XRD was performed on an Angstrom Advanced Inc. ADX-2700 powder diffractometer with a monochromatic CuK_α1_ X-ray line and a modified Anton Paar ALTK-450 VT stage. Color images shown in Figs. 2, 4 and 5 were recorded using a Thorlabs color CCD camera (DCU224C) mounted on an inverted stage microscope illuminated using a single polarizer.

### Mixture formation

DACLC mixtures were made by weighing out the correct molar ratio of components, and then physically mixing with a spatula prior to melting with a heat gun. The resulting mixture was iteratively corrected using ^1^H NMR until integration of the respective donor and acceptor peaks gave a ratio of 1.00 to 1.00 (± 0.02). Laser writing tests were performed on samples sandwiched between substrates (usually clean glass slides), melted, and then allowed to cool at 2 °C/min to room temperature.

### N1,N5-dihexylnaphthalene-1,5-diamine (DAN, D2)

In a round bottom flask, naphthalene-1,5-diamine (1.00 g, 6.32 mmol, Aldrich), 50 mL of acetone, and K_2_CO_3_ (5.24 g, 37.93 mmol, Fluka) was added. The reaction was refluxed and stirred for 30 min. Then 1-bromohexane (6.78 g, 41.09 mmol, Aldrich) was added and continued to reflux for 72 h. Acetone was removed in vacuo and the crude product was purified by column chromatography in DCM:hexanes (7:3 Hex:DCM). The eluting solvent was removed in vacuo, and the product was further purified by crystallization in isopropanol to yield light-purple needle-like crystals of **1** (0.6 g, 30% yield). ^1^H NMR (CDCl_3_, 400 MHz) δ 0.90 (t, J = 7.88, 6H), 1.27–1.37 (m, 8H), 1.37–1.45 (m, 4H), 1.63–1.72 (m, 4H), 3.13–3.20 (m, 4H), δ 5.76 (t, J = 4.72, 2H), δ 6.45 (d, J = 7.04, 2H), δ 7.17 (t, J = 9.4, 2H), δ 7.31 (d, 2H). ^13^C NMR (CDCl_3_, 400 MHz) 14.03 (2C), 22.72 (2C), 27.12 (2C), 29.48 (2C), 31.66 (2C), 44.32 (2C), 104.30 (2C), 108.47 (2C), 123.93 (2C), 125.46 (2C), 144.27 (2C). Expected mass: 326.27, ESI–MS (negative-ion) measured mass: 326.3.

### N,N-dioctyl-naphthalenediimide (NDI, A)

1,4,5,8-Naphthalenetetracarboxylic dianhydride (1.0 g, 3.4 mmol) was placed into a round bottom flask and suspended in isopropanol (80 ml). A mixture of 1-aminooctane (1.6 g, 12.4 mmol), TEA (1.3 g, 13 mmol), and isopropanol (30 ml) was slowly added and the solution was allowed to stir at room temperature for 30 min, and then heated at reflux for 16 h. The solution was allowed to cool to room temperature and the resulting precipitate was filtered and recrystallized in isopropanol to yield **2** (1.6 g, 94% yield) as off-white crystals. ^1^H NMR (CDCl_3_, 400 MHz) *δ* 8.66 (s, 4H), 4.14 (t, *J* = 7.5 Hz, 4H), 1.82 (p, *J* = 7.2 Hz, 4H), 1.55–1.20 (m, 10H), 0.91 (t, *J* = 6.9 Hz, 6H) ppm. Expected mass: 490.28, ESI–MS (negative-ion) measured mass: 490.3.

### Laser patterning of DACLC films

Each DACLC thin film was fabricated by filling a glass cell, comprised of a glass coverslip and microscope slide separated by 20 µm silica beads, via capillary action at 175 °C. Patterns were subsequently written into DACLC films using the NanoScribe GmbH Photonic Professional GT 3D printer equipped with a 20 × Zeiss EC Epiplan-Neofluar 0.50 NA objective and adjusting the power, scan speed and hatch angle of the scanning beam to control the degree and direction of columnar alignment in each pixel. Isotropic regions were written using a 50 mm/s laser scan speed and 40–60% of maximum laser power. Depending on the size of the pixel/scanning region, anisotropic areas were written using a 1.5–3.5 mm/s scan speed and 10–12% of maximum laser power, with the resultant polarization direction perpendicular to the hatching direction (thus parallel to the direction of the thermal gradient produced by the laser). Optical microscope images were taken using a Nikon Eclipse TI equipped with a single polarizer. Image analysis was performed using custom scripts written in MATLAB (Natick, MA, USA, MathWorks; Version R2018a, 9.4.0.813654; License Number: STUDENT). Annotated scripts are available at https://github.com/howwallace/reczekj-et-al-2020.git.

### Three-image splining fit of written DACLC regions (θ_w_)

LPL transmittance values for three images taken at differing θ_LPL_ values of written DACLC regions (Supplementary Fig. 2) were used to confirm or determine θ_w_. Values for θ_LPL_ of 0°, 45°, and 90° were chosen to maximize the range of phase differential intensity, although any three θ_LPL_ angles with > 15° spacing can be used to attain similar accuracy. The θ_w_ for each region is determined by a four-parameter sinusoidal fit of Eq. 3. This technique, termed “splining,” allows for accurate fitting of sinusoidal curves with only three points (the values of I_obs_ from the three ∆ θ_LPL_ images) and known period (180°).^S2, S3^ The four-parameter sinusoidal fits from only there LPL images were compared to least-squares regressions of data from all 36 LPL images (Fig. 2d) and found to yield negligibly different values for θ_w_.

Note that the parameters of k’ and b’ from Eq. 3 are effectively “fitting constants” in this splining method. They are determined independently for each DACLC region in the fitting process, and then inherently normalized in the analysis of the total image. That is to say, the lowest intensity value of a global image analysis is defined as b′ = 0, and the value of k′ is proportional to the effective dichroic ratio between the min and max I_obs_.

## Supplementary information

Supplementary file1
