# Supplementary material for: Direct-write orientation of charge-transfer liquid crystals enables polarization-based coding and encryption
Source: Sci Rep. 2020 Sep 18;10:15352. doi: 10.1038/s41598-020-72037-z (PMC7501303; doi:10.1038/s41598-020-72037-z)
Supplement: Supplementary file 1 — Supplementary file1 [file 41598_2020_72037_MOESM1_ESM.docx]

*Supplementary Information*

for

**Direct-write orientation of charge-transfer liquid crystals enables polarization-based coding and encryption​**

*Madeline Van Winkle^1^, Harper O. W. Wallace^1^, Niquana Smith^1^, Andrew T. Pomerene^2^, Michael G. Wood^2^, Bryan Kaehr* ^,2,3^, Joseph J. Reczek*^,1^*

^1^Department of Chemistry, Denison University, Granville, Ohio, 43023, USA

^2^Sandia National Laboratories, Albuquerque, New Mexico, 87185, USA

^3^Center for Integrated Nanotechnologies, Sandia National Laboratories, Albuquerque, NM, 87185, USA

*E-mail: bjkaehr@sandia.gov; reczekj@denison.edu

Contents:

Detailed Methods Page 2 - 3

Figures S1 - S4 Page 4 - 6

Tables S1, S2 Page 6 - 8

^1^H NMR spectra Page 9 - 11

References Page 11

**Detailed Methods**

**General Considerations.** All commercial reagents and solvents were obtained from Sigma-Aldrich or Fischer Scientific and were used as purchased without additional purification. Compounds **1** and **2** were synthesized following published procedures.^S1^ ^1^H and ^13^C NMR spectra were collected using a Bruker Avance 400 MHz spectrometer at 25 ºC. LPL microscopy was performed using an Olympus BX51TRF microscope and accessories from McCrone Microscopes in transmission mode on a Linkam large area thermal stage. Images were captured with a PAXCAM 3 camera. Thermal analysis data by DSC was collected on a Q20 instrument with an RSC cooling system from TA instruments. UV/Vis spectroscopy was performed on a JAZ-PX spectrophotometer from Ocean Optics. LPL UV/Vis spectroscopy was performed by fitting the JAZ-PX spectrophotometer directly to the Olympus BX51TRF microscope ocular. Variable temperature powder XRD was performed on an Angstrom Advanced Inc. ADX-2700 powder diffractometer with a monochromatic CuK_α1_ X-ray line and a modified Anton Paar ALTK-450 VT stage. Color images shown in Figures 2, 4 and 5 were recorded using a Thorlabs color CCD camera (DCU224C) mounted on an inverted stage microscope illuminated using a single polarizer.

**Mixture formation**. DACLC mixtures were made by weighing out the correct molar ratio of components, and then physically mixing with a spatula prior to melting with a heat gun. The resulting mixture was iteratively corrected using ^1^H NMR until integration of the respective donor and acceptor peaks gave a ratio of 1.00 to 1.00 (± 0.02). Laser writing tests were performed on samples sandwiched between substrates (usually clean glass slides), melted, and then allowed to cool at 2 ˚C/min to room temperature.

**N1,N5-dihexylnaphthalene-1,5-diamine (DAN, D2).** In a round bottom flask, naphthalene-1,5-diamine (1.00 g, 6.32 mmol, Aldrich), 50 mL of acetone, and K_2_CO_3_ (5.24 g, 37.93 mmol, Fluka) was added. The reaction was refluxed and stirred for 30 minutes. Then 1-bromohexane (6.78 g, 41.09 mmol, Aldrich) was added and continued to reflux for 72 hours. Acetone was removed *in vacuo* and the crude product was purified by column chromatography in DCM:hexanes (7:3 Hex:DCM). The eluting solvent was removed *in vacuo,* and the product was further purified by crystallization in isopropanol to yield light-purple needle-like crystals of **1** (0.6 g, 30% yield). ^1^H NMR (CDCl_3_, 400 MHz) δ 0.90 (t, J=7.88, 6H), 1.27-1.37 (m, 8H), 1.37-1.45 (m, 4H), 1.63-1.72 (m, 4H), 3.13-3.20 (m, 4H), δ 5.76 (t, J=4.72, 2H), δ 6.45 (d, J=7.04, 2H), δ 7.17 (t, J=9.4, 2H), δ 7.31 (d, 2H). ^13^C NMR (CDCl_3_, 400 MHz) 14.03 (2C), 22.72 (2C), 27.12 (2C), 29.48 (2C), 31.66 (2C), 44.32 (2C), 104.30 (2C), 108.47 (2C), 123.93 (2C), 125.46 (2C), 144.27 (2C). Expected mass: 326.27, ESI –MS (negative-ion) measured mass: 326.3.

**N,N-dioctyl-naphthalenediimide (NDI, A).** 1,4,5,8-Naphthalenetetracarboxylic dianhydride (1.0g, 3.4mmol) was placed into a round bottom flask and suspended in isopropanol (80 ml). A mixture of 1-aminooctane (1.6g, 12.4 mmol), TEA (1.3g, 13 mmol), and isopropanol (30ml) was slowly added and the solution was allowed to stir at room temperature for 30 min, and then heated at reflux for 16 hours. The solution was allowed to cool to room temperature and the resulting precipitate was filtered and recrystallized in isopropanol to yield **2** (1.6g, 94% yield) as off-white crystals. ^1^H NMR (CDCl_3_, 400 MHz) *δ* 8.66 (s, 4H), 4.14 (t, *J* = 7.5 Hz, 4H), 1.82 (p, *J* = 7.2 Hz, 4H), 1.55-1.20 (m, 10H), 0.91 (t, *J* = 6.9 Hz, 6H) ppm. Expected mass: 490.28, ESI –MS (negative-ion) measured mass: 490.3.

**Laser patterning of DACLC films**. Each DACLC thin film was fabricated by filling a glass cell, comprised of a glass coverslip and microscope slide separated by 20 µm silica beads, via capillary action at 175°C. Patterns were subsequently written into DACLC films using the NanoScribe GmbH Photonic Professional GT 3D printer equipped with a 20x Zeiss EC Epiplan-Neofluar 0.50 NA objective and adjusting the power, scan speed and hatch angle of the scanning beam to control the degree and direction of columnar alignment in each pixel. Isotropic regions were written using a 50 mm/s laser scan speed and 40–60% of maximum laser power. Depending on the size of the pixel/scanning region, anisotropic areas were written using a 1.5–3.5 mm/s scan speed and 10–12% of maximum laser power, with the resultant polarization direction perpendicular to the hatching direction (thus parallel to the direction of the thermal gradient produced by the laser). Optical microscope images were taken using a Nikon Eclipse TI equipped with a single polarizer. Image analysis was performed using custom scripts written in MATLAB (Natick, MA, USA, MathWorks; Version R2018a, 9.4.0.813654; License Number: STUDENT). Annotated scripts are available at <https://github.com/howwallace/reczekj-et-al-2020.git>.

**Three-Image splining fit of written DACLC regions** (θ_w_). LPL transmittance values for three images taken at differing θ_LPL_ values of written DACLC regions (Supplementary Fig. 2) were used to confirm or determine θ_w_. Values for θ_LPL_ of 0, 45, and 90° were chosen to maximize the range of phase differential intensity, although any three θ_LPL_ angles with >15˚ spacing can be used to attain similar accuracy. The θ_w_ for each region is determined by a four-parameter sinusoidal fit of **Equation 3**. This technique, termed “splining,” allows for accurate fitting of sinusoidal curves with only three points (the values of I_obs_ from the three ∆ θ_LPL_ images) and known period (180˚).^S2, S3^ The four-parameter sinusoidal fits from only there LPL images were compared to least-squares regressions of data from all 36 LPL images (Figure 2d) and found to yield negligibly different values for θ_w_.

Note that the parameters of k’ and b’ from **Equation 3** are effectively “fitting constants” in this splining method. They are determined independently for each DACLC region in the fitting process, and then inherently normalized in the analysis of the total image. That is to say, the lowest intensity value of a global image analysis is defined as b’ = 0, and the value of k’ is proportional to the effective dichroic ratio between the min and max I_obs_.


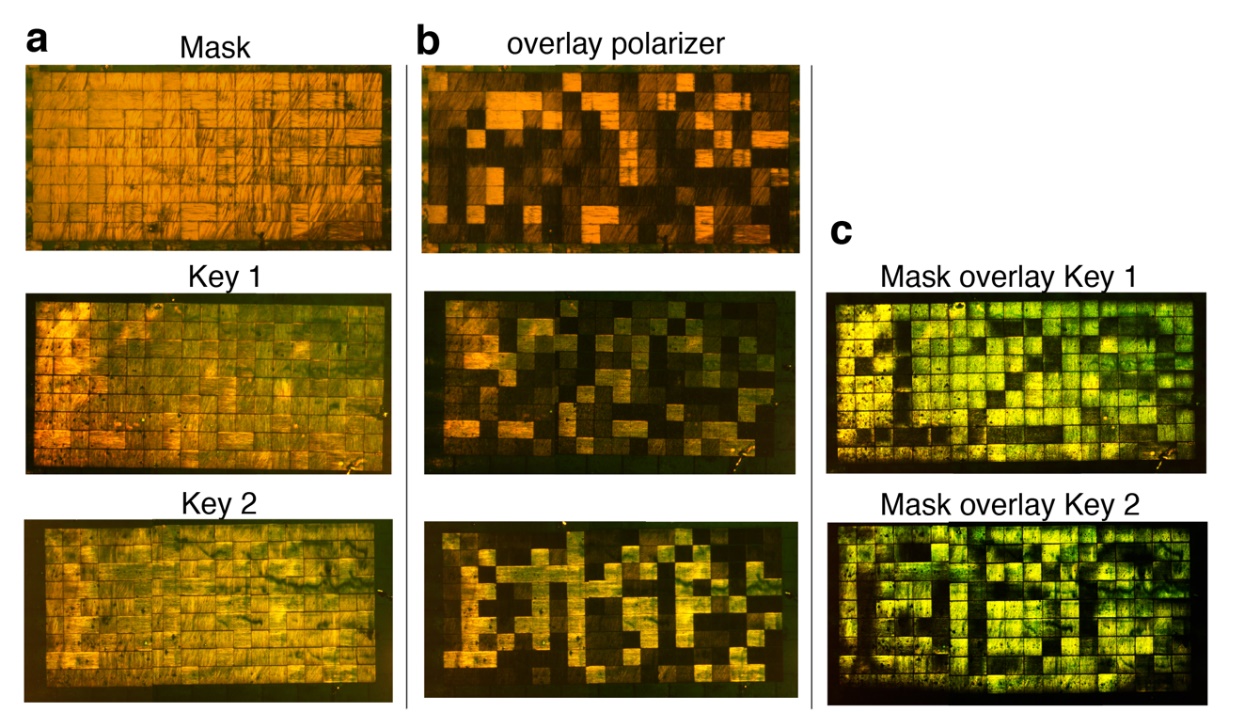


**Supplementary Fig. 1**. Expansion of Mask-Key overlay showing a single mask can be used with multiple “keys” to display independent information. a) Mask and keys viewed under microscope with no polarizing filter. b) Mask and keys viewed under a single polarizing filter. c) A single Mask overlaid with “Key 1” and then “Key 2” to reveal the independent messages “123” and “ABC” respectively.

**Supplementary Fig. 2.** Example image used in intensity/contrast analysis of LPL-transmittance through 36 independently aligned DACLC film regions. a) Original grayscale image of LPL-exposed grid. b) Illustration of regions used in analysis: Pixel coordinate of 50 x 50 µm aligned regions are outlined in red, and [(97 µm)^2^ – (74 µm)^2^ = (63 µm)^2^] isotropic borders are outlined in blue. These regions are identified using draggable rectangle tool (find_region_of_interest.m). Average grayscale values calculated for each aligned region (red squares) are normalized using the corresponding immediate bordering isotropic background (blue outlined border) to determine internally consistent transmittance intensity from LPL image data (process_img.m). This process is carried out with three images (θ_LPL_ = 0, 45, and 90°) for the splining process to determine the write angle as described above.


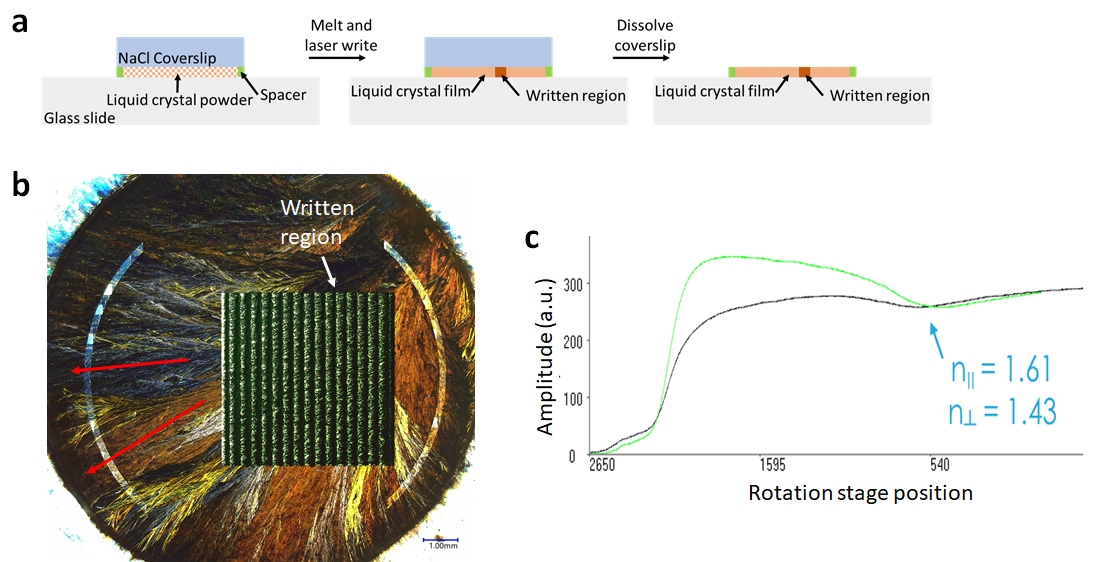


**Supplementary Fig. 3.** Refractive index of DACLC films. a) Schematic showing sample fabrication in order to measure refractive index through directly exposure to interrogating light. b) Optical image of the sample measured. The red lines indicate the direction of LC columns in the bulk sample. c) Prism coupling measurements in the aligned LC film. Dips in the measured amplitude in these curves correspond to coupled light from the prism into the film. By rotating the sample 90° and repeating the measurement, the refractive indices at a wavelength of 635 nm was measured to be 1.61 and 1.43 when the incident light is parallel and perpendicular to the LC columns, respectively. Additional measurements in the isotropic laser patterned region (written region shown in panel b) of the LC film gave an index of 1.43 with no change when rotating the sample.


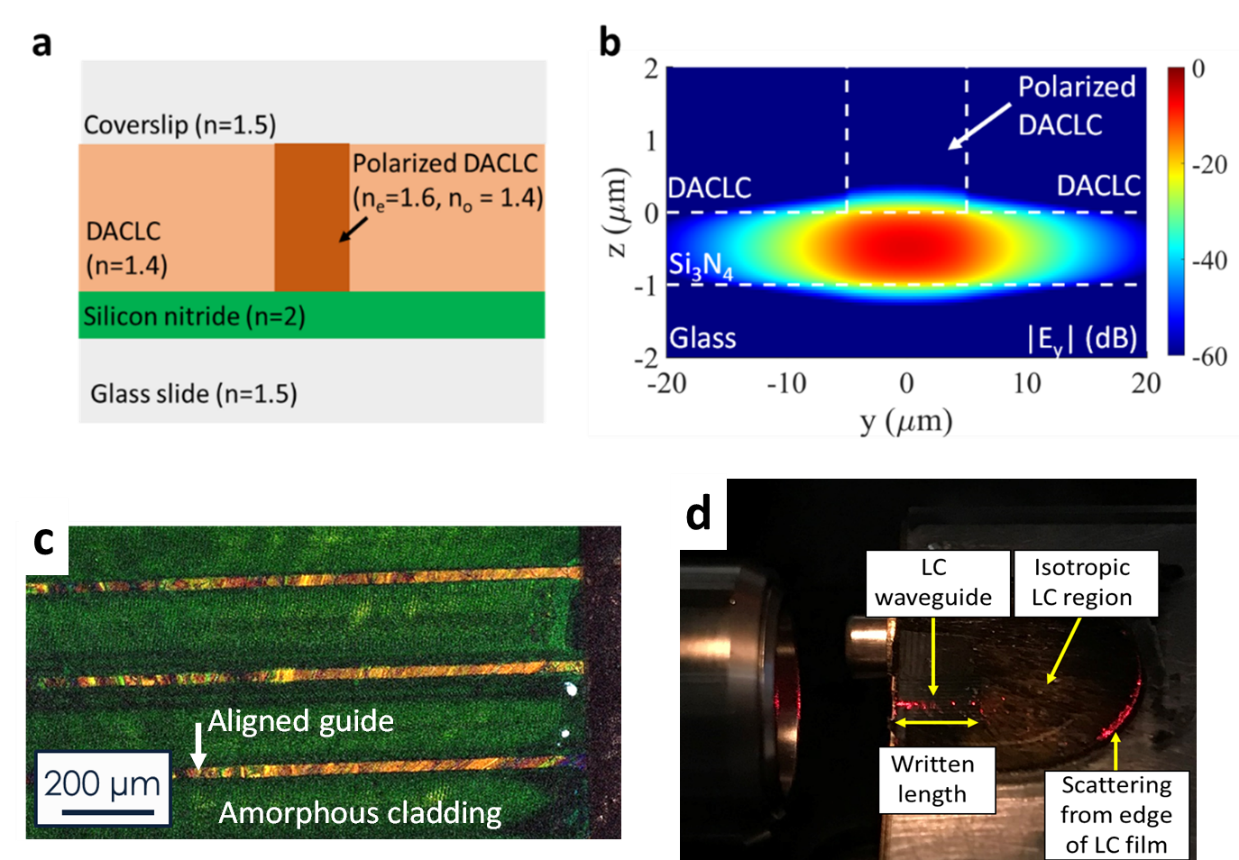


**Supplementary Fig. 4.** Modeling a DACLC waveguide. a) Strip-loaded waveguide geometry used for waveguide modeling. b) Numerical solution for the strip-loaded waveguide mode in the LC film modeled using a commercial finite element method (FEM) package (Lumerical MODE). White lines indicate edges of the boundaries of the different materials used in the simulation. (c) An array of 50 µm wide waveguides whereby the amorphous cladding is laser scanned to confine the higher index core. (d) A 635 nm laser is coupled into an LC waveguide (from the left) resulting in scattering at the far edge of the film which indicates the possibility of DACLC-based waveguides.

**Supplementary Table 1.** Dyad encoding scheme. Note that each character value corresponds redundantly to six dyads, and that each of six dyads corresponds uniquely to a single character value. Dyads used in the example message, “ALICE’S_MESSAGE_TO_BOB” (Figure 5d), are shown in bold.

| Character value | Dyad 1 | Dyad 2 | Dyad 3 | Dyad 4 | Dyad 5 | Dyad 6 |
| --- | --- | --- | --- | --- | --- | --- |
| A | 20, 140 | 10, 140 | **0, 60** | 40, 130 | 100, 10 | **110, 20** |
| B | 10, 90 | **140, 50** | 70, 10 | 80, 0 | 90, 10 | 170, 70 |
| C | 50, 140 | **160, 70** | 90, 130 | 120, 10 | 10, 110 | 170, 140 |
| D | 60, 140 | 50, 80 | 70, 50 | 160, 50 | 130, 90 | 150, 100 |
| E | **50, 70** | 140, 140 | **50, 90** | 80, 120 | **10, 100** | 40, 30 |
| F | 10, 130 | 20, 30 | 10, 30 | 140, 80 | 160, 140 | 90, 120 |
| G | 140, 120 | 60, 120 | 150, 0 | 120, 110 | 80, 150 | **130, 10** |
| H | 10, 150 | 80, 70 | 50, 10 | 160, 120 | 90, 70 | 160, 150 |
| I | 170, 80 | **130, 80** | 170, 170 | 170, 20 | 110, 40 | 40, 0 |
| J | 50, 0 | 80, 10 | 60, 100 | 100, 90 | 170, 120 | 60, 30 |
| K | 70, 0 | 130, 60 | 90, 20 | 160, 170 | 10, 160 | 0, 50 |
| L | 120, 30 | 100, 130 | **20, 40** | 120, 100 | 60, 110 | 20, 100 |
| M | 30, 0 | 80, 30 | 0, 20 | 50, 50 | 90, 30 | 170, 130 |
| N | 70, 90 | 140, 160 | 30, 130 | 0, 150 | 70, 130 | 70, 80 |
| O | 80, 80 | 150, 50 | **150, 140** | 120, 80 | 0, 10 | **90, 110** |
| P | 30, 140 | 170, 30 | 60, 60 | 0, 100 | 50, 100 | 20, 170 |
| Q | 100, 30 | 110, 170 | 40, 40 | 150, 90 | 110, 120 | 130, 0 |
| R | 30, 50 | 120, 0 | 120, 20 | 60, 40 | 110, 150 | 140, 10 |
| S | 120, 170 | 160, 90 | 150, 40 | **140, 170** | **160, 0** | 130, 110 |
| T | 40, 10 | 40, 50 | 30, 170 | 30, 70 | 30, 150 | 120, 120 |
| U | 70, 20 | 140, 130 | 120, 150 | 130, 70 | 50, 160 | 70, 70 |
| V | 110, 130 | 100, 150 | 120, 40 | 50, 120 | 20, 10 | 130, 30 |
| W | 110, 60 | 70, 160 | 40, 80 | 90, 90 | 140, 60 | 100, 60 |
| X | 80, 140 | 110, 30 | 40, 160 | 20, 50 | 80, 170 | 170, 150 |
| Y | 80, 60 | 0, 160 | 130, 40 | 120, 60 | 70, 40 | 170, 100 |
| Z | 110, 140 | 160, 110 | 40, 70 | 150, 130 | 0, 130 | 100, 120 |
| _A | 130, 120 | 140, 20 | 80, 110 | 20, 90 | 160, 60 | 80, 100 |
| _B | 10, 170 | 170, 110 | 60, 70 | 40, 90 | 170, 160 | **30, 160** |
| _C | 150, 20 | 30, 10 | 150, 120 | 160, 100 | 30, 80 | 130, 50 |
| _D | 100, 160 | 100, 170 | 70, 170 | 170, 50 | 140, 0 | 160, 160 |
| _E | 50, 130 | 50, 20 | 110, 50 | 60, 80 | 130, 170 | 0, 70 |
| _F | 50, 60 | 90, 60 | 160, 10 | 130, 130 | 70, 110 | 40, 100 |
| _G | 30, 40 | 110, 110 | 120, 140 | 50, 150 | 120, 160 | 150, 150 |
| _H | 40, 60 | 150, 160 | 70, 150 | 110, 100 | 140, 30 | 100, 70 |
| _I | 0, 40 | 130, 20 | 20, 110 | 90, 160 | 120, 130 | 90, 140 |
| _J | 150, 10 | 50, 170 | 20, 160 | 30, 30 | 10, 20 | 170, 40 |
| _K | 10, 60 | 140, 40 | 20, 150 | 140, 150 | 160, 130 | 100, 40 |
| _L | 60, 0 | 20, 0 | 150, 80 | 30, 90 | 10, 120 | 20, 120 |
| _M | 100, 0 | **30, 120** | 40, 110 | 70, 120 | 110, 90 | 40, 170 |
| _N | 90, 150 | 10, 80 | 150, 110 | 100, 20 | 60, 10 | 30, 60 |
| _O | 40, 150 | 20, 130 | 40, 140 | 10, 70 | 20, 60 | 60, 150 |
| _P | 100, 110 | 150, 30 | 50, 30 | 100, 80 | 80, 160 | 10, 0 |
| _Q | 50, 110 | 110, 10 | 20, 70 | 140, 110 | 70, 30 | 0, 140 |
| _R | 0, 0 | 60, 160 | 170, 10 | 110, 0 | 170, 0 | 90, 100 |
| _S | 110, 160 | 10, 40 | 70, 140 | 150, 170 | 60, 170 | 70, 100 |
| _T | 70, 60 | 160, 20 | 100, 50 | **80, 40** | 120, 70 | 20, 80 |
| _U | 140, 100 | 60, 50 | 0, 30 | 0, 170 | 40, 120 | 120, 50 |
| _V | 130, 100 | 40, 20 | 100, 100 | 10, 50 | 50, 40 | 110, 70 |
| _W | 90, 40 | 80, 90 | 20, 20 | 90, 50 | 0, 120 | 0, 80 |
| _X | 30, 110 | 10, 10 | 30, 20 | 60, 130 | 130, 160 | 0, 90 |
| _Y | 0, 110 | 170, 60 | 160, 80 | 120, 90 | 140, 70 | 60, 90 |
| _Z | 110, 80 | 140, 90 | 160, 40 | 30, 100 | 150, 60 | 80, 130 |
| 'S | 100, 140 | **60, 20** | 90, 170 | 170, 90 | 130, 150 | 150, 70 |
| 'T | 90, 80 | 130, 140 | 80, 20 | 160, 30 | 80, 50 | 90, 0 |

**Supplementary Table 2.** Encoded message readout accuracy using base-10 and base-19 encoding schemes. Using a base-10 encoding scheme (such that exact readout angle is rounded to the nearest 20° increment; in this case, because the encoding scheme is defined in 10° increments, readout is considered to be “correct” if it falls within 10° of the actual written angle), readout of the encoded message is perfectly accurate. Using a base-20 encoding scheme (such that exact readout angle is rounded to the nearest 10° increment), readout is near-perfect, with one error in 36 readings. Redundancies built into the cipher (Supplementary Table 1) were not leveraged to obscure any erroneous readout.

Written Angle (°)

| 0 | 20 | 130 | 160 | 50 | 60 |
| --- | --- | --- | --- | --- | --- |
| 60 | 40 | 80 | 70 | 90 | 20 |
| 30 | 10 | 140 | 160 | 110 | 130 |
| 120 | 100 | 170 | 0 | 20 | 10 |
| 50 | 80 | 150 | 30 | 90 | 140 |
| 70 | 40 | 140 | 160 | 110 | 50 |

Angle Readout (°)

| 0.3 | 18.3 | 127.0 | 165.6 | 48.1 | 58.6 |
| --- | --- | --- | --- | --- | --- |
| 59.4 | 41.0 | 79.0 | 68.0 | 88.1 | 18.1 |
| 26.3 | 8.2 | 141.5 | 161.5 | 109.8 | 129.1 |
| 117.3 | 98.5 | 174.5 | 179.8 | 17.9 | 8.2 |
| 46.9 | 77.5 | 149.0 | 26.5 | 91.2 | 143.5 |
| 72.4 | 39.4 | 140.3 | 164.8 | 106.8 | 47.8 |

**^1^H NMR spectrum of compounds synthesized in this work**


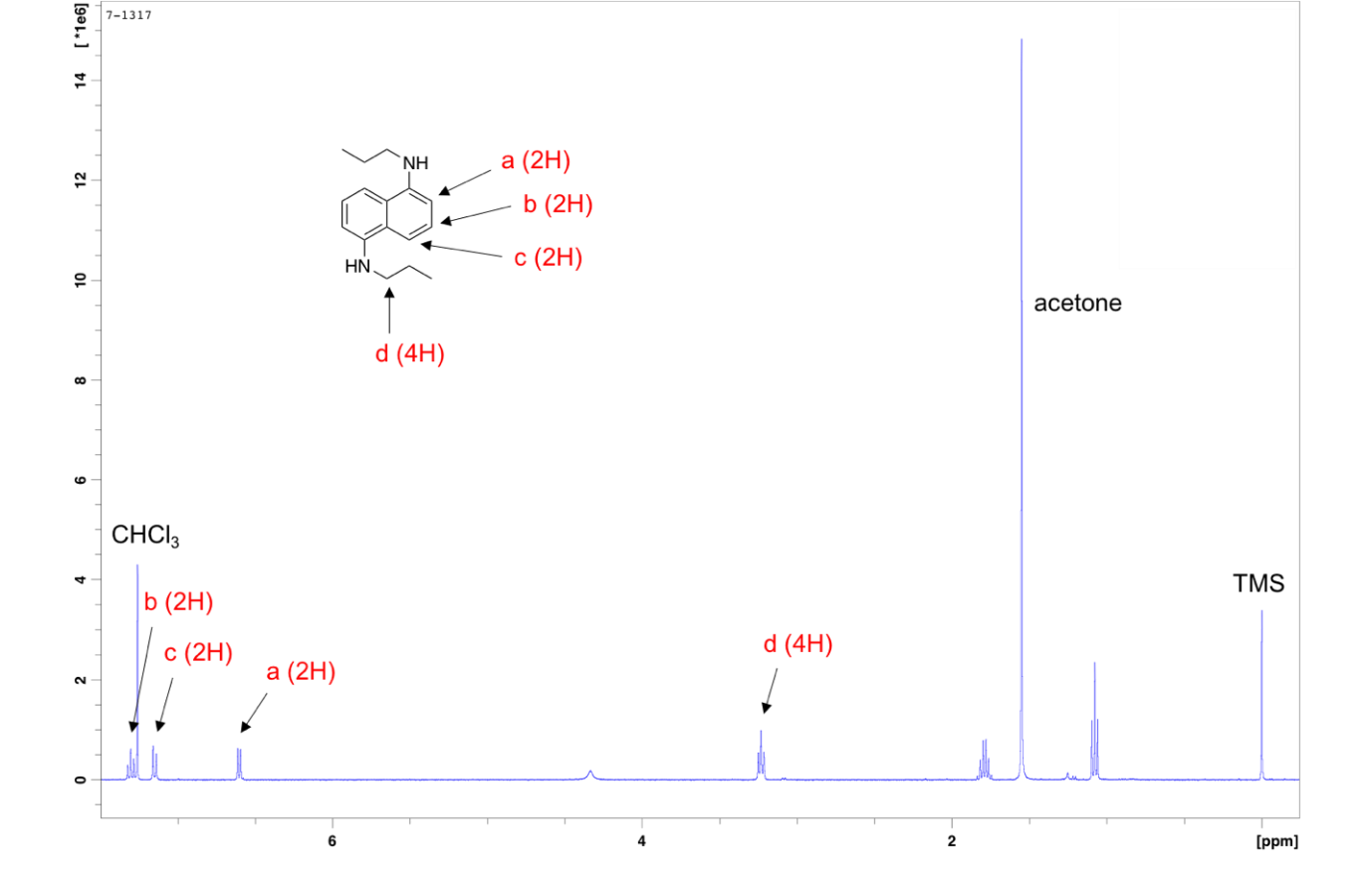


400 MHz ^1^H NMR spectrum in CDCl_3_ of molecule D components **A** and D**1.**


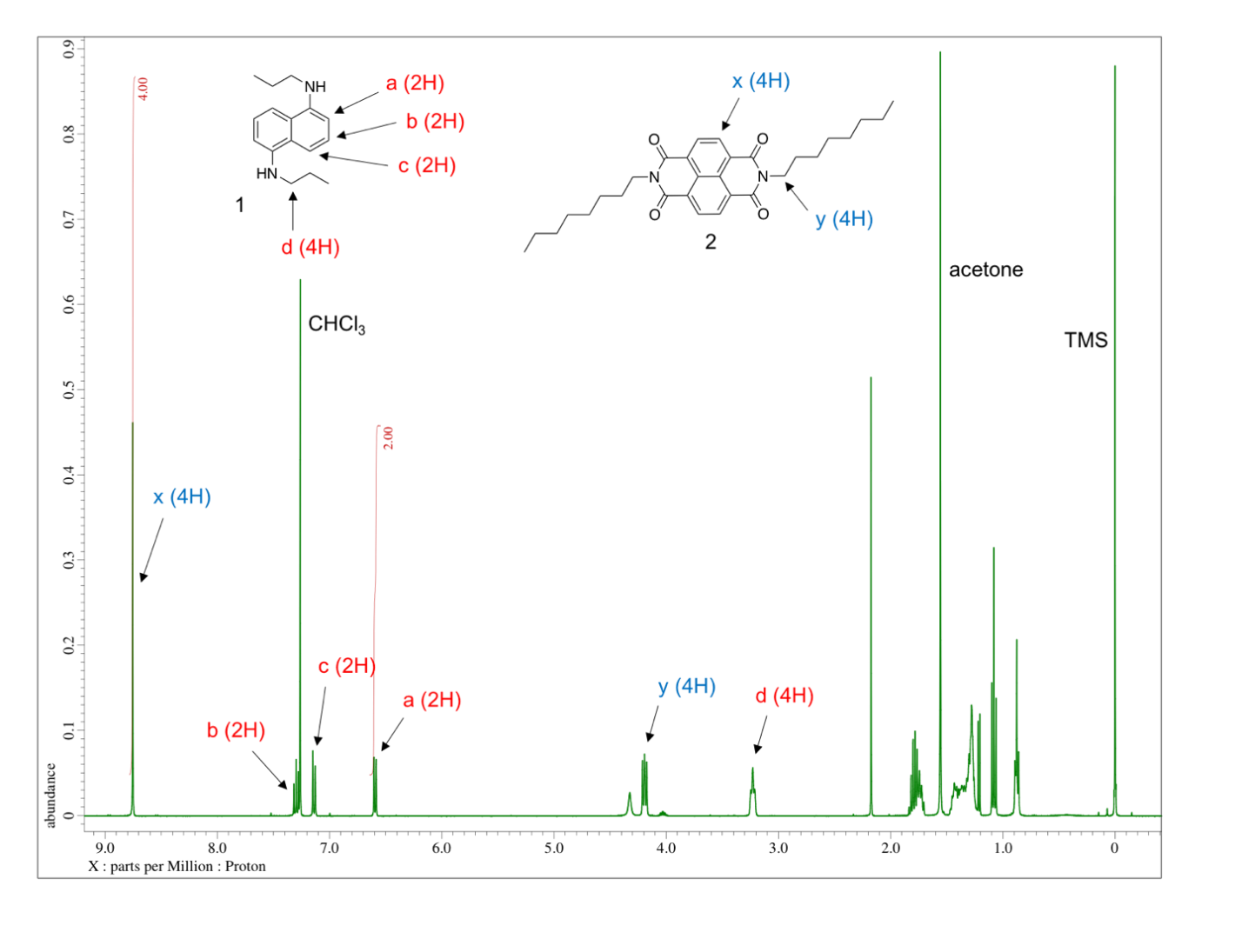


400 MHz ^1^H NMR spectrum in CDCl_3_ of the 1:1 molar ratio DACLC mixture of components **A** and D**1.**


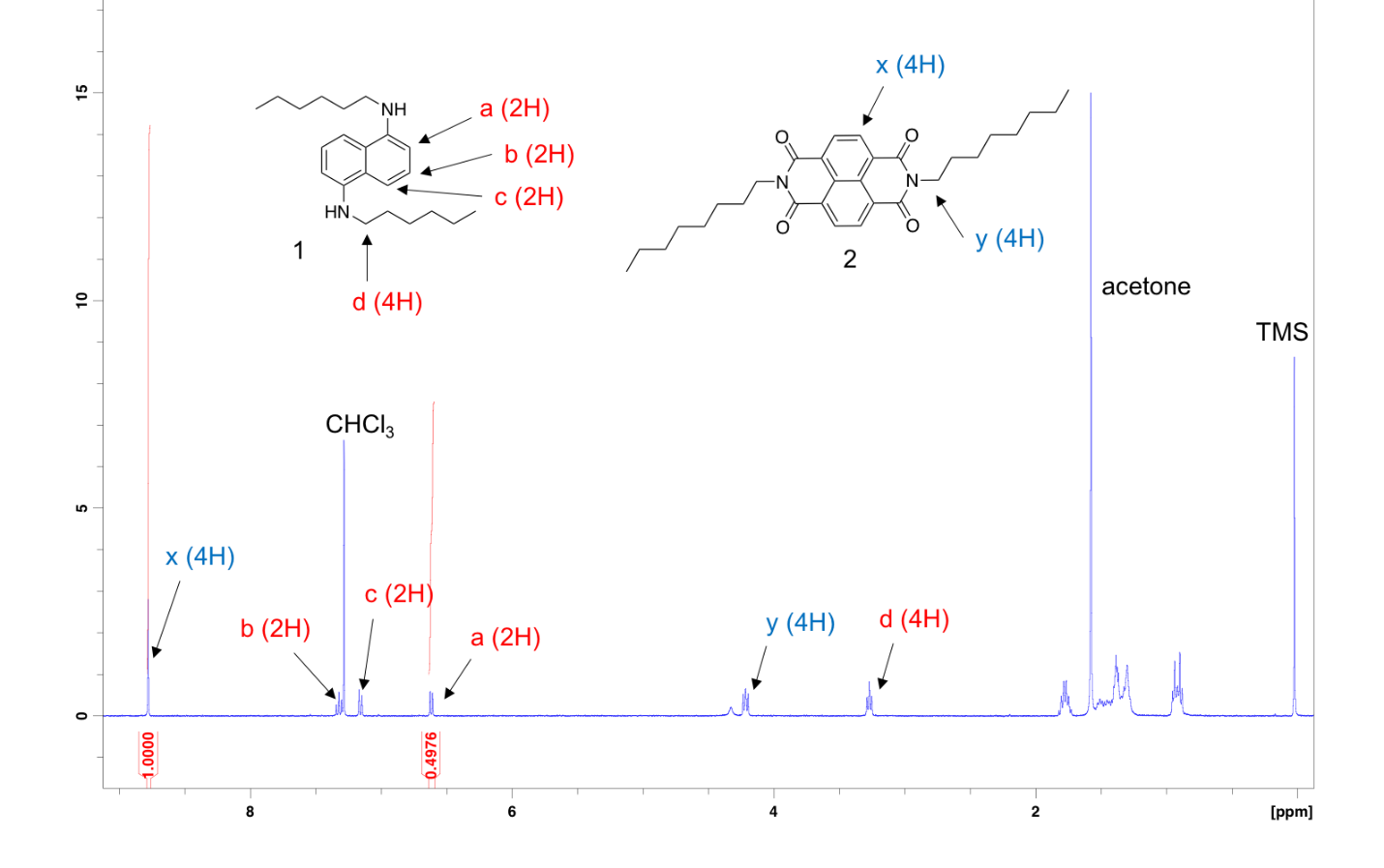


400 MHz ^1^H NMR spectrum in CDCl_3_ of the 1:1 molar ratio DACLC mixture of components **A** and D**2**.

**References for Supporting Information:**

S1. Leight, K. R.; Esarey, B. E.; Murray, A. E.; Reczek, J. J. Modular and Predictable Tuning of Absorption Properties in Aromatic Donor-Acceptor Materials *Chem. Mater.* *24*, 3318-3328 (2012).

S2. Alegria, F. C. Bias of amplitude estimation using three-parameter sine fitting in the presence of additive noise. *Measurement, 42*(5), 748–756 (2009).

S3. Basis for 3-image spinning of region alignment: Ramos, P. M., Da Silva, F., & Serra, A. C. Improving sine-fitting algorithms for amplitude and phase measurements. In *XVII IMEKO World Congress, Dubrovnik, Croatia*, 614–619 (2003).
